# Iodide of Ethyl in Asthma

**Published:** 1880-07

**Authors:** Daniel R. Brower


### Clinical Reports.

ARTICLE V.

*Iodide of Ethyl in Asthma.*

I have recently had a very satisfactory experience with this remedy in an obstinate case of asthma.

The patient is a youth about fifteen years old, who inherits instability of nervous action from both parents. He has had obstinate attacks for six years past, especially during the spring and summer months.

The only complete relief he has heretofore had has been by change of residence. He has tried about all the remedies that have been suggested, such as nitrate of amyl, chloral, morphia, bromide, belladonna and galvanism, without benefit. Partial relief was obtained for some time by smoking a portion of the following combination, which in some cases has acted well:

℞ Dracenti radi. pulv................................℥j

Stramonii foliæ pulv................................℥j

Lobeliæ pulv.........................................ʒvj

Potassii nitratis pulv..............................℥ss

M.

In the attack that commenced this spring this recipe seems to have been of but little service. I therefore ordered him, as recommended by Prof. Lee of Paris, inhalation of the iodide of ethyl. The preparation used was made by Nesrck of Darmstadt and imported by E. H. Sargent & Co. of this city.

After several trials we found the effective dose to be six drops. This relieved the paroxysms as if by magic and no unpleasant symptoms followed its use. The only new sensation there seems to have been experienced was occasionally a slight sense of numbness in the feet and hands. Under its daily use the intervals between the paroxysms have grown longer and the severity of the attacks has been relieved.

It may be well to add that for some time past, previous to the use of the iodide of ethyl, I had been giving him iodide of potassium with tonics, but the surprising effects upon the paroxysms was clearly due to this new remedy for asthma.

DANIEL R. BROWER, M.D.

---

